# Serum uric acid, disease severity and outcomes in COVID-19

**DOI:** 10.1186/s13054-021-03616-3

**Published:** 2021-06-14

**Authors:** Inès Dufour, Alexis Werion, Leila Belkhir, Anastazja Wisniewska, Marie Perrot, Julien De Greef, Gregory Schmit, Jean Cyr Yombi, Xavier Wittebole, Pierre-François Laterre, Michel Jadoul, Ludovic Gérard, Johann Morelle, the CUSL COVID-19 Research Group, Christophe Beauloye, Christine Collienne, Mélanie Dechamps, Florence Dupriez, Philippe Hantson, Luc-Marie Jacquet, Benoit Kabamba, Fatima Larbaoui, Virginie Montiel, Andrea Penaloza, Lucie Pothen, Hector Rodriguez-Villalobos, Anais Scohy, Maximilien Thoma, Olivier Van Caeneghem, Halil Yildiz

**Affiliations:** 1 Division of Nephrology, Cliniques universitaires Saint-Luc, 1200 Brussels, Belgium; 2 Division of Infectious Diseases, Cliniques universitaires Saint-Luc, Brussels, Belgium; 3 Department of Intensive Care Medicine, Cliniques universitaires Saint-Luc, 1200 Brussels, Belgium; 4 Department of Pathology, Cliniques universitaires Saint-Luc, Brussels, Belgium; 5 Institut de Recherche Expérimentale et Clinique, UCLouvain, Brussels, Belgium

**Keywords:** SARS-CoV-2, Acute respiratory distress syndrome, Mechanical ventilation, Proximal tubule, Hypouricemia

## Abstract

**Background:**

The severity of coronavirus disease 2019 (COVID-19) is highly variable between individuals, ranging from asymptomatic infection to critical disease with acute respiratory distress syndrome requiring mechanical ventilation. Such variability stresses the need for novel biomarkers associated with disease outcome. As SARS-CoV-2 infection causes a kidney proximal tubule dysfunction with urinary loss of uric acid, we hypothesized that low serum levels of uric acid (hypouricemia) may be associated with severity and outcome of COVID-19.

**Methods:**

In a retrospective study using two independent cohorts, we investigated and validated the prevalence, kinetics and clinical correlates of hypouricemia among patients hospitalized with COVID-19 to a large academic hospital in Brussels, Belgium. Survival analyses using Cox regression and a competing risk approach assessed the time to mechanical ventilation and/or death. Confocal microscopy assessed the expression of urate transporter URAT1 in kidney proximal tubule cells from patients who died from COVID-19.

**Results:**

The discovery and validation cohorts included 192 and 325 patients hospitalized with COVID-19, respectively. Out of the 517 patients, 274 (53%) had severe and 92 (18%) critical COVID-19. In both cohorts, the prevalence of hypouricemia increased from 6% upon admission to 20% within the first days of hospitalization for COVID-19, contrasting with a very rare occurrence (< 1%) before hospitalization for COVID-19. During a median (interquartile range) follow-up of 148 days (50–168), 61 (12%) patients required mechanical ventilation and 93 (18%) died. In both cohorts considered separately and in pooled analyses, low serum levels of uric acid were strongly associated with disease severity (linear trend, *P* < 0.001) and with progression to death and respiratory failure requiring mechanical ventilation in Cox (adjusted hazard ratio 5.3, 95% confidence interval 3.6–7.8, *P* < 0.001) or competing risks (adjusted hazard ratio 20.8, 95% confidence interval 10.4–41.4, *P* < 0.001) models. At the structural level, kidneys from patients with COVID-19 showed a major reduction in urate transporter URAT1 expression in the brush border of proximal tubules.

**Conclusions:**

Among patients with COVID-19 requiring hospitalization, low serum levels of uric acid are common and associate with disease severity and with progression to respiratory failure requiring invasive mechanical ventilation.

**Supplementary Information:**

The online version contains supplementary material available at 10.1186/s13054-021-03616-3.

## Background

SARS-CoV-2 is the novel coronavirus that causes the pandemic of coronavirus disease 2019 (COVID-19) [1]. COVID-19 primarily affects the respiratory tract, with a broad spectrum of clinical manifestations, ranging from asymptomatic infection to severe pneumonia, respiratory failure and the need for mechanical ventilation in 10–20% of hospitalized patients [2–4]. Life-threatening COVID-19 is characterized by an acute respiratory distress syndrome, which typically aggravates during in a second phase of the disease driven by an excessive host response [5, 6]. Variability in disease presentation and progression stresses the need for easily available and reliable biomarkers to identify patients at risk of the most severe forms and to provide optimal, personalized care to the individual patient [5].

COVID-19 is also characterized by extra-pulmonary manifestations involving the gastrointestinal tract, the neurological and cardiovascular systems, and the kidneys [7]. We recently demonstrated that SARS-CoV-2 causes a specific dysfunction of the kidney proximal tubule, as attested by the presence of low molecular weight proteinuria, neutral aminoaciduria and defective tubular handling of uric acid [8]. In a cohort of 49 patients with specific urinalysis, inappropriate uricosuria was associated with disease severity and the need for mechanical ventilation [8]. Whether the use of widely available serum uric acid levels can be used as a surrogate for the tubular defect and as a biomarker of disease severity and outcome in COVID-19 has not been assessed.

Here we used two large and independent cohorts of consecutive patients hospitalized with SARS-CoV-2 infection to investigate and validate the prevalence, kinetics and clinical correlates of hypouricemia in COVID-19.

## Materials and methods

### Study design and participants

**Fig. 1:**
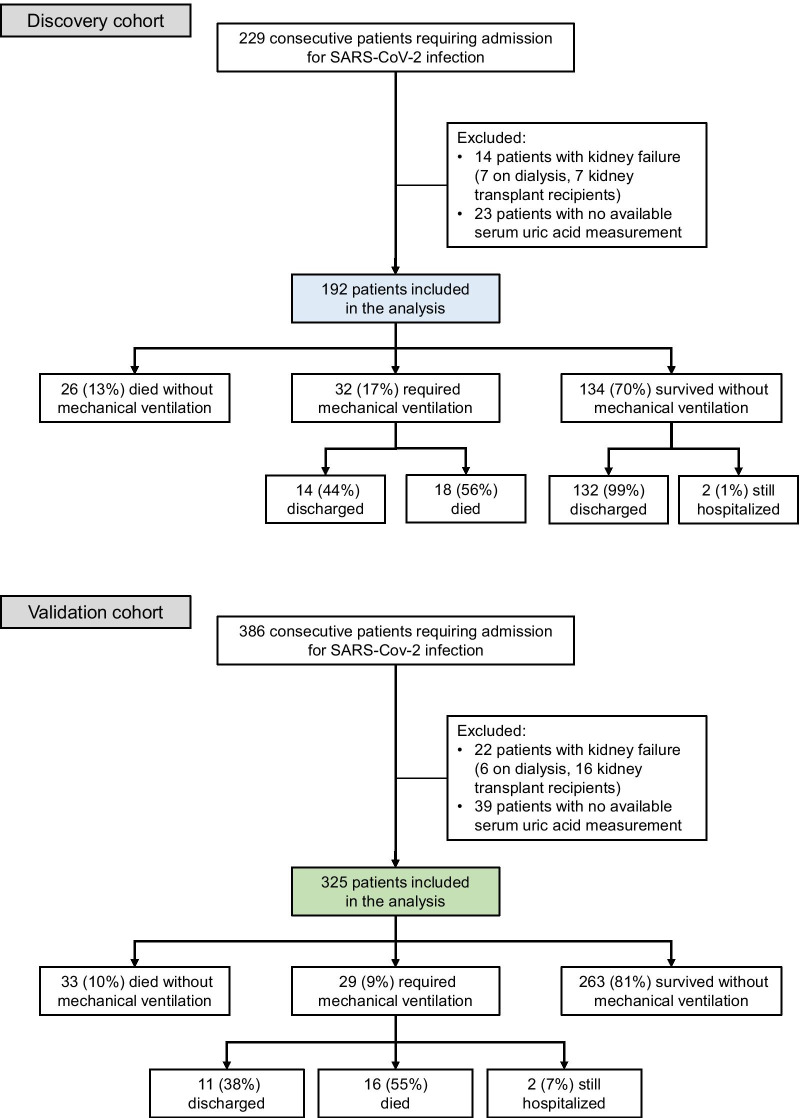
Flowchart of the study, including discovery and validation cohorts

This retrospective study included two independent, discovery and validation cohorts of consecutive adult patients admitted to the Cliniques universitaires Saint- Luc, Brussels, Belgium, with a diagnosis of SARS-CoV-2 infection during the first (February 23, 2020 to April 18, 2020) and second (September 21, 2020 to November 21, 2020) waves of the pandemic. Patients on chronic kidney replacement therapy (hemodialysis, peritoneal dialysis or kidney transplantation) or for whom serum uric acid levels were not available were excluded. COVID-19 diagnosis was based on the detection of SARS-CoV-2 by real-time reverse transcription polymerase chain reaction on nasopharyngeal swab or broncho-alveolar lavage. The standard treatment for patients hospitalized for COVID-19 during the first wave (discovery cohort) included hydroxychloroquine 400 mg b.i.d. on the first hospital day and then 200 mg b.i.d. for four additional days in patients without contraindication, as recommended at the time of the study by the Belgian COVID-19 interim guidelines. The standard treatment for severe and critical patients hospitalized during the second wave (validation cohort) consisted of oral dexamethasone at a dose of 6 mg once daily for up to 10 days [9]. Enteral nutrition using Fresubin HP Energy Fibre (Fresenius Kabi, Schelle, Belgium) was initiated early (< 48 h) in all patients admitted to the ICU and as needed in those hospitalized in a non-ICU COVID-19 unit. We aimed for a target of 25–35 kcal/kg/day and 1.2–1.3 g/kg protein equivalents per day. All patients admitted to the ICU could be fully fed through the enteral route, and we did not use parenteral nutrition in any of them. Crystalloid solutions (Hartmann, alternatively 0.9% saline) were used. Patients were followed until death or end of study follow-up (July 31, 2020 for the discovery cohort and April 11, 2021 for the validation cohort). The flowcharts are presented in Fig. 1. A subset of 49 patients who underwent specific urinalyses to screen for the presence of proximal dysfunction were previously reported [8]. The twenty-three individuals from the discovery cohort for whom no uric acid level was available included (i) elderly patients with severe comorbidities, major therapeutic restrictions and a high mortality rate, and (ii) patients with mild or moderate disease and a very short hospital length of stay (Additional file 1: Table S1).

The study was conducted in accordance with the World Medical Association’s Declaration of Helsinki, the Belgian law related to experiments in humans dated May 7, 2004, the General Data Protection Regulation 2016/679 and the Belgian law of July 30, 2018, regarding the protection of personal data. The Ethical Review Board of Cliniques universitaires Saint-Luc/UCLouvain approved the study and waived the requirement to obtain informed consent based on the retrospective observational design of the study.

### Data collection and definitions

The following data were extracted from electronic medical records: demographics, symptoms at admission, vital signs, biological and imaging data, and outcome. Information about the use of the following drugs, known to interfere with uric acid metabolism, was also collected: allopurinol, febuxostat, fenofibrate, angiotensin receptor blockers and trimethoprim-sulfamethoxazole.

Laboratory measurements were performed on an automated Roche Cobas 8000 analyzer, equipped with modules ISE, c702, c502 and e602 (Roche diagnostics, Rotkreuz, ZG, Switzerland), or a UC3500 automated urine analyzer (Sysmex). Biological data were obtained at admission, and the worst (lowest or highest) levels were also recorded for some variables. Serum uric acid level was collected from medical records at the time of admission, at the time of first level under 2.5 mg/dl (if any) as a cutoff previously used for the study in SARS-CoV-1 [10] and at lowest level in both cohorts. Serum uric acid level was systematically assessed upon admission. Monitoring during hospitalization was systematic and performed on a daily basis among those admitted to the ICU, and left to the discretion of the physician in those on the general ward. In the small subset of patients requiring kidney replacement therapy, uric acid levels were only recorded before initiation of dialysis. In the discovery cohort, serum uric acid level was also collected at discharge from hospital, as well as one to twelve months before admission for COVID-19 in a subset of patients (*n* = 122) for whom these data were available. Inappropriate uricosuria was defined as a fractional excretion of uric acid > 10% in the presence of hypouricemia [8, 10]. In a previous report, detailed urinalyses showed that, in patients with COVID-19, hypouricemia is commonly associated with inappropriate uricosuria (fractional excretion > 10% in 18/20 [90%], and 7% and 8% in the two remaining individuals), supporting defective tubular handling and enhanced urinary losses of uric acid [8]. The definition of acute kidney injury was adapted from the KDIGO guidelines [11, 12]. As urine output was not recorded digitally in most patients (i.e., those admitted to a general ward), the definition of AKI was restricted to changes in serum creatinine, as previously done [13]. The term ‘mechanical ventilation’ refers to intubation followed by invasive mechanical ventilation.

Classification of COVID-19 severity was based on the adaptation of the Novel Coronavirus Pneumonia Diagnosis and Treatment Guidance [14]. Mild disease was defined as SARS-CoV-2 infection with no evidence of pneumonia on thoracic computed tomography (CT) scan; moderate disease, as clinical symptoms associated with dyspnea and radiological findings of pneumonia on thoracic CT scan, and requiring a maximum of 3 L/min of oxygen, stable for at least the following 24 h; severe disease, as respiratory distress requiring more than 3 L/min of oxygen and no other organ failure, stable for at least the following 24 h; and critical COVID-19, as respiratory failure requiring mechanical ventilation, shock and/or other organ failure requiring intensive care unit admission. Disease severity was defined as the worst severity score during hospital stay and also evaluated upon admission to assess progression during the course of the disease.

### SARS-CoV-2 polymerase chain reaction

SARS-CoV-2 RNA detection was performed using COVID-19 genesig® Real-Time RT-PCR assay (Primerdesign Ltd, Chandler’s Ford, United Kingdom) in a LightCycler 480 instrument (Roche Diagnostics, Mannheim, Germany). Primers and probe of this assay target the RNA-dependent RNA polymerase gene. A test with a cycle threshold less than 40 was considered positive.

### Immunostaining

Immunofluorescence was performed on kidney samples obtained from autopsies of five patients who died from COVID-19 and three control patients. Paraffin blocks were sectioned into consecutive 5-μm-thick slices on Superfrost Plus glass slides (Thermo Fisher Scientific, Merelbeke, Belgium). Before staining, slides were deparaffinized in decreasing concentrations of ethanol and antigen retrieval was performed by incubating in sodium citrate buffer (1.8% 0.1 M citric acid, 8.2% 0.1 M sodium citrate, in distillated water, pH 6.0) in a water bath for 30 min. The sections were blocked with phosphate-buffered saline containing 5% BSA and incubated for 1 h with primary antibodies. Rabbit anti-human SLC22A12/URAT1 (HPA024575, Sigma-Aldrich, Saint-Louis, MO) and rabbit anti-human AQP1 (ab2219, Millipore) were used in this study. After 3 phosphate-buffered saline rinses, fluorophore-conjugated Alexa secondary antibodies (Invitrogen, Carlsbad, CA) were applied for 30 min. Negative controls were performed by omitting the primary antibody. Sections were subsequently mounted in ProLong Gold DAPI Antifade reagent (Invitrogen) and analyzed on a Zeiss LSM800 confocal microscope (Carl Zeiss, Jena, Germany), using × 20/0.8 Plan-Apochromat (Carl Zeiss). Quantitative image analysis was performed using Zen 2 (blue edition) software (Carl Zeiss) by randomly selecting 5 visual fields per each slide that included at least three to five proximal tubules, using constant setting parameters (i.e., pinhole, laser power, and offset gain and detector amplification below pixel saturation).

### Statistical analysis

Results are presented as means ± SD or median [interquartile range (IQR)] for continuous variables and as numbers and proportions for categorical variables. Continuous variables were expressed in their natural units without standardization. Comparisons between groups were performed using unpaired t-test, Mann–Whitney, Kruskal–Wallis, or Chi-square test, as appropriate. Kinetics of serum uric acid was assessed using a mixed effects model taking into account repeated measures.

Survival analyses were performed using Kaplan–Meier estimates, Cox proportional hazard regressions and a competing risk model. Kaplan–Meier estimates and Cox proportional hazard regressions assessed the time to mechanical ventilation or death, and a Fine and Gray model was applied for the time to mechanical ventilation taking into account the competing risks of death or discharge. Multivariate analyses included the following pre-specified covariates: age, gender and baseline biological parameters (CRP, LDH, total lymphocyte count) in a first model and the same parameters and biological parameters of disease severity (highest CRP and LDH levels) in a second model. Nadir lymphocyte count was not included in Model 2 because of collinearity with baseline lymphocyte count. Collinearity between variables was quantified using variance inflation factors, and variance inflation factors > 10 suggested excessive correlation between variables.

Statistical analyses were performed using GraphPad Prism (version 8.0) or Stata (version 16.0) softwares. All tests were two-tailed, and a *P *value < 0.05 was considered significant.

## Results

### Transient hypouricemia is highly prevalent in hospitalized patients with COVID-19

Our study included a total of 517 patients hospitalized for COVID-19, recruited during the first (discovery cohort, *n* = 192) or second (validation cohort, *n* = 325) waves of the pandemic in Belgium (Fig. 1). Considering the whole study population, median age [IQR] was 65 years [54–76], and 60% were males (Table 1). Comorbidities included hypertension, cardiovascular disease and diabetes in 229 (44%), 152 (29%) and 125 (24%) individuals, respectively. Thirty-five (7%) patients were treated with allopurinol or febuxostat; 10 (2%), with fenofibrate; and 165 (32%), with inhibitors of the renin angiotensin system. Based on the Diagnosis and Treatment Protocol for Novel Coronavirus Pneumonia [14], 38 (7%) patients had a mild; 113 (22%) moderate; and 274 (53%) severe COVID-19; the remaining 92 (18%) had a disease classified as critical.

**Table 1:** Baseline characteristics of patients in discovery and validation cohorts

Demographics and comorbidities	Pooled data *n* = 517	Discovery cohort *n* = 192	Validation cohort *n* = 325
Age, median (IQR), years	65 (54–76)	65 (55–79)	65 (54–74)
Male gender—no. (%)	311 (60)	106 (55)	205 (63)
Ethnicity—no. (%)			
Caucasian	451 (87)	162 (84)	289 (89)
Sub-Saharan African	56 (11)	27 (14)	29 (9)
Other	10 (2)	3 (2)	7 (2)
Cardiovascular disease—no. (%)	152 (29)	44 (23)	108 (33)
Chronic kidney disease—no. (%)	75 (15)	39 (20)	36 (11)
Hypertension—no. (%)	229 (44)	97 (51)	132 (41)
Diabetes—no. (%)	125 (24)	46 (24)	79 (24)
Medications—no. (%)			
Allopurinol or febuxostat	35 (7)	11 (6)	24 (7)
Fenofibrate	10 (2)	3 (2)	7 (2)
Trimethoprim-sulfamethoxazole	20 (4)	6 (3)	14 (4)
Angiotensin receptor blocker	81 (16)	30 (16)	51 (16)
ACE inhibitor	84 (16)	39 (20)	45 (14)
*Lab tests at admission*			
hsCRP, median (IQR), mg/l	74 (39–132)	74 (38–130)	76 (40–132)
Creatinine, median (IQR), mg/dl	1.0 (0.8–1.2)	1.0 (0.8–1.2)	1.0 (0.8–1.2)
eGFR, median (IQR), ml/min/1.73 m^2^	76 (54–92)	72 (50–85)	80 (58–94)
Serum uric acid, median (IQR), mg/dl	4.6 (3.6–6.1)	4.8 (3.7–6.2)	4.6 (3.6–6.1)
LDH, median (IQR), IU/l	330 (262–424)	349 (270–449)	325 (260–409)
Lymphocytes, median (IQR), µl^−1^	890 (610–1200)	830 (590–1150)	900 (620–1220)
*Disease severity*			
Classification—no. (%)			
Mild	38 (7)	14 (7)	24 (7)
Moderate	113 (22)	35 (18)	78 (24)
Severe	274 (53)	99 (52)	175 (54)
Critical	92 (18)	44 (23)	48 (15)
Peak hsCRP, median (IQR), mg/l	122 (65–209)	138 (72–262)	113 (60–188)
Nadir lymph. count, median (IQR), µl^−1^	700 (420–1010)	650 (400–945)	720 (460–1040)
Peak LDH, median (IQR), IU/l	411 (313–530)	438 (325–580)	403 (305–514)
*Outcome*			
Follow-up, median (IQR), days	148 (50–168)	53 (37–60)	165 (150–173)
ICU admission—no. (%)	108 (21)	57 (30)	51 (16)
Death—no. (%)	93 (18)	44 (23)	49 (15)
Mechanical ventilation—no. (%)	61 (12)	32 (17)	29 (9)
Acute kidney injury—no. (%)	58 (11)	26 (14)	32 (10)
Kidney replacement therapy—no. (%)	16 (3)	6 (3)	10 (3)
Hospital LOS, median (IQR), days	10 (5–16)	12 (7–18)	8 (5–14)

Continuous variables are expressed as median and interquartile range (IQR) and categorical variables as numbers (no.) and percentages (%)

ACE, angiotensin converting enzyme; eGFR, CKD-EPI estimated glomerular filtration rate; hsCRP, highly sensitive C-reactive protein; LDH, lactate dehydrogenase; LOS, length of stay

**Fig. 2:**
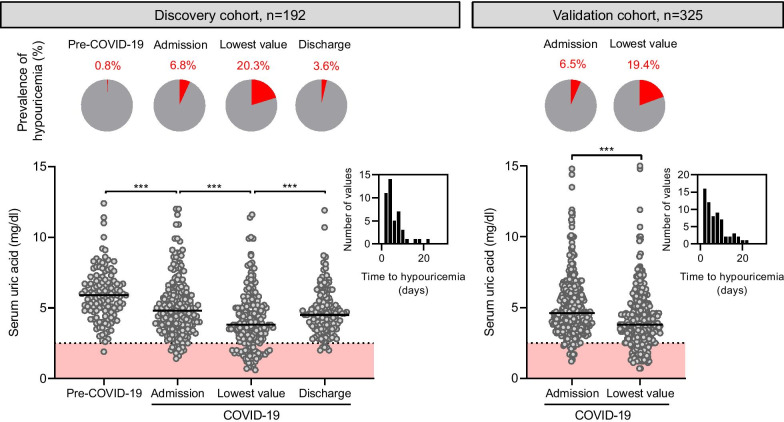
Prevalence and kinetics of low serum uric acid levels among patients hospitalized with COVID-19, in discovery and validation cohorts. **a** In the discovery cohort, the prevalence of hypouricemia was 1/122 (0.8%) under basal conditions, 13/192 (6.8%) at admission, 39/192 (20.3%) at the lowest uric acid level and 5/140 (3.6%) at discharge. **b** In the validation cohort, the prevalence of hypouricemia increased from 21/325 (6.5%) upon admission to 63/325 (19.4%) during hospitalization. Circles represent individual values and black lines medians. Histograms show the distribution of time from admission to onset of hypouricemia in both cohorts. *P* values calculated using a mixed-effects model considering repeated measures in individual patients. ***P < 0.001

We first examined the prevalence and timing of hypouricemia in patients hospitalized for COVID-19. In the discovery cohort, the prevalence of hypouricemia increased from 6.8% (13/192) upon admission to 20.3% (39/192) within the first days of hospitalization and then dropped to 3.6% (5/140) at discharge (Fig. 2). In contrast with the high prevalence during hospitalization for COVID-19, low serum levels of uric acid were very uncommon in the same cohort, before SARS-CoV-2 infection. Indeed, in the 122 individuals from the discovery cohort for whom serum uric acid levels before admission (median of 79 days [31–192]) were available, the prevalence of hypouricemia was only 0.8% (1/122) during the pre-COVID-19 era. These observations were replicated in the validation cohort, in which the prevalence of low serum uric acid levels increased from 6.5% (21/325) upon admission to 19.4% (63/325) during hospitalization (Fig. 2). The occurrence of hypouricemia was independent of the use of drugs interfering with uric acid production, nephrotoxic medications, treatment received for COVID-19, or viral load (Additional file 1: Table S2).

Altogether, these observations show that serum uric acid levels decrease early during hospitalization for COVID-19, leading to severe hypouricemia in a significant proportion of patients, which then reverses among survivors.

### Low serum levels of uric acid associate with disease severity and outcome in COVID-19

To assess the clinical relevance of low serum levels of uric acid in patients hospitalized with COVID-19, we compared disease severity and outcome of patients with versus without hypouricemia.

**Fig. 3:**
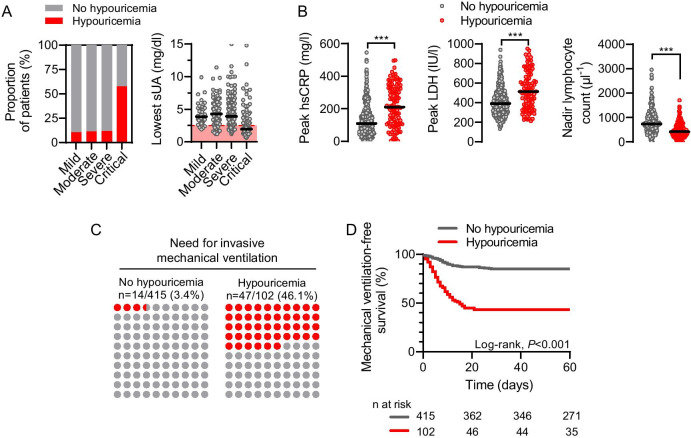
Low serum uric acid levels associate with disease severity and outcome in COVID-19 (pooled data from discovery and validation cohorts). **a** Proportion of patients with hypouricemia (bar graph) and lowest serum uric acid level (scatter plots with median) according to COVID-19 severity. Circles represent individual values and black lines are medians (one-way ANOVA and linear trend, *P* = 0.002). **b** Comparison of peak values of highly sensitive C-reactive protein (hsCRP) and lactate dehydrogenase (LDH), and nadir of lymphocytes in patients with or without hypouricemia. Circles represent individual values and lines are medians. ***P < 0.001 using Mann–Whitney test. **c** Proportion of patients requiring mechanical ventilation, according to the presence (red dots) or not (gray dots) of hypouricemia. **d** Survival analyses with Kaplan–Meier curves showing time to mechanical ventilation or death (log-rank test, *P* < 0.001)

In both discovery and validation cohorts, the presence of hypouricemia strongly correlated with disease severity and with established biological parameters of severity, including hsCRP, LDH and nadir lymphocyte count (Fig. 3, Table 2). Considering pooled data from discovery and validation cohorts, the proportion of patients with hypouricemia was 17/151 (11%) among those with mild or moderate; 32/275 (12%) with severe; and 53/92 (58%) with critical disease (Fig. 3a; Table 2). Patients with critical COVID-19 also had lower serum uric acid levels during hospitalization (median [IQR] 2.0 [1.4–3.9] vs. 3.9 [3.0–5.2], 4.3 [3.3–5.2] and 3.9 [3.1–5.3] mg/dl in patients with severe, moderate and mild disease, respectively, linear trend, *P* < 0.001) (Fig. 3a). The presence of hypouricemia not only associated with severity of COVID-19, but also with progression of severity during the course of the disease, including 5 (13%) patients who progressed from moderate to severe; 7 (18%) from moderate to critical; and 9 (23%) from severe to critical disease, in the discovery cohort. In the group without hypouricemia, 18 (12%) with moderate progressed to severe, 2 (1.3%) with moderate progressed to critical; and 11 (7%) progressed from severe to critical (*P *value = 0.006) (Additional file 1: Fig. S1).

**Table 2:** Disease severity and outcomes of patients without or with hypouricemia

	Discovery cohort, *n* = 192			Validation cohort, *n* = 325		
	No hypouricemia, *n* = 153	Hypouricemia, *n* = 39	*P*	No hypouricemia, *n* = 262	Hypouricemia, *n* = 63	*P*
*Disease severity*						
Classification—no. (%)			< 0.001			< 0.001
Mild	13 (9)	1 (3)		21 (8)	3 (5)	
Moderate	32 (21)	3 (8)		68 (26)	10 (16)	
Severe	88 (58)	11 (28)		154 (59)	21 (33)	
Critical	20 (13)	24 (62)		19 (7)	29 (46)	
Peak hsCRP, median (IQR), mg/l	123 (65–221)	266 (114–349)	< 0.001	102 (54–161)	205 (106–302)	< 0.001
Nadir lymph. count, median (IQR), µl^−1^	690 (440–1000)	410 (210–770)	< 0.001	785 (560–1110)	440 (280–640)	< 0.001
Peak LDH, median (IQR), IU/l	404 (318–515)	559 (456–733)	< 0.001	384 (293–479)	514 (343–746)	< 0.001
*Outcomes*						
Death—no. (%)	31 (20)	13 (33)	0.1	27 (10)	22 (35)	< 0.001
Mechanical ventilation—no. (%)	10 (7)	22 (56)	< 0.001	4 (2)	25 (40)	< 0.001
Acute kidney injury—no. (%)	17 (11)	5 (13)	0.8	13 (5)	19 (31)	< 0.001
Kidney replacement therapy—no. (%)	4 (3)	2 (5)	0.4	3 (1)	7 (11)	< 0.001
Hospital LOS, median (IQR), days	11 (6–16)	20 (14–39)	< 0.001	8 (4–12)	15 (8–38)	< 0.001

Continuous variables are expressed as median and interquartile range (IQR) and categorical variables as numbers (no.) and percentages (%)

hsCRP, highly sensitive C-reactive protein; lymph., lymphocytes; LDH, lactate dehydrogenase; LOS, length of stay

Patients who developed hypouricemia also showed higher levels of CRP (211 [110–326] vs. 110 [59–174] mg/l, *P* < 0.001); higher peak of LDH (535 [374–733] vs. 392 [306–492], *P* < 0.001); and lower nadir count of lymphocytes (420 [260–660] vs. 740 [490–1060], *P* < 0.001), than patients without hypouricemia (Fig. 3b; Table 2).

Overall, patients were followed for a median [IQR] of 148 days [50–168]. During follow-up, 108 (21%) patients were admitted to the ICU, 59 (11%) died without mechanical ventilation; 61 (12%) were started on mechanical ventilation; and 397 (77%) were discharged without mechanical ventilation (Fig. 1; Table 2). In both cohorts and in the pooled analysis, there was a strong association between hypouricemia and progression to respiratory failure requiring mechanical ventilation (pooled data: 47/102 [46%] vs. 14/415 [3%], *P* < 0.001, in patients with and without hypouricemia, respectively) (Table 2; Fig. 3c and d). The prevalence of hypouricemia was higher in participants admitted to the ICU vs. those who were not (53/108 [49%] vs. 49/409 [12%], *P* < 0.001).

The kinetics of serum uric acid levels in patients who required mechanical ventilation was thoroughly assessed in the discovery cohort. Serum uric acid levels started to decrease before the start of mechanical ventilation; reached a nadir (1.7 mg/dl [1.15–2.80]) 48–72 h after intubation; then started to re-increase in the majority of patients (21/29, 72%) (Additional file 1: Fig. S2A). In this subgroup, time from admission to lowest level of serum uric acid (median [IQR] 7 days [5–10] was similar to time to the worst value of other established biomarkers, including peak LDH (6 days [2–10]), peak CRP (7 days [4–9]) and nadir lymphocyte count (8 days [4–11]) (*P* > 0.05 for all) (Additional file 1: Fig. S2B). Median time [IQR] to hypouricemia was 4.5 days [3–7] and, in patients requiring mechanical ventilation, 45% (10/22) had developed severe hypouricemia before mechanical ventilation had to be initiated (Additional file 1: Fig. S2A). To rule out any impact from volume resuscitation on the dilution of serum uric acid, we thoroughly assessed fluid balance in the subset of patients from the discovery cohort developing hypouricemia while in ICU (*n* = 25), where this information was digitally recorded. In this subset of patients, the amount of fluid infused over the 2 days before the lowest value of uric acid was minimal, with a median [IQR] positive fluid balance of 186 ml per day [− 58, 1050]. The absence of any significant impact from excessive volume resuscitation on serum uric acid levels is also supported by (i) a decrease in serum uric acid levels already upon admission, as compared with baseline values obtained for a large subset (*n* = 122) of patients; (ii) plasma sodium concentration and serum osmolality in the normal range, with a median of 139 mmol/l [137–142] and 286 mOsm/kg [283–301], respectively, at the time of the lowest level of uric acid recorded during hospitalization.

Survival analyses using Kaplan–Meier estimates showed reduced mechanical ventilation-free survival among patients with versus those without hypouricemia (log-rank, *P* < 0.001) (Fig. 3d; Table 3). In each cohort considered separately, and in pooled data, time-to-event analyses using Cox regressions supported the significant association between hypouricemia and higher risk of invasive mechanical ventilation or death (pooled data: hazard ratio [HR] 5.1; 95% confidence interval [CI] 3.6–7.4, *P* < 0.001) and showed the association was independent from age, gender and biological parameters of disease severity (adjusted HR 5.6, 95% CI 3.8–8.3, *P* < 0.001). A Fine and Gray model considering mechanical ventilation as the primary endpoint and death or discharge from hospital as competing events confirmed the association between hypouricemia and the need of mechanical ventilation, both in unadjusted and unadjusted models (sHR 18.1, 95% CI 9.7–33.8, *P* < 0.001; adjusted sHR 18.8, 95% CI 9.3–38.1, *P* < 0.001) (Table 3). The sensitivity and specificity of hypouricemia as a biomarker for COVID-19 requiring mechanical ventilation were 77% and 88%, respectively, and the likelihood ratio, 6.4. Of note, the proportion of patients who developed hypouricemia (23/27 [85%] vs. 24/34 [71%), *P* = 0.18) was similar among survivors and non-survivors of critical COVID-19 requiring mechanical ventilation.

**Table 3:** Cox and competing risks regressions for time to invasive mechanical ventilation according to the presence of hypouricemia

	Univariate			Multivariate—model 1			Multivariate—model 2		
	HR	95% CI	*P*	Adj. HR	95% CI	*P*	Adj. HR	95% CI	*P*
*Cox (mechanical ventilation or death)*									
No hypouricemia	1.0 (ref.)	–	–	1.0 (ref.)	–	–	1.0 (ref.)	–	–
Hypouricemia (discovery)	3.9	2.3–6.7	< 0.001	6.6	3.6–12.1	< 0.001	3.1	1.6–6.1	0.001
Hypouricemia (validation)	6.7	4.1–11.1	< 0.001	6.2	3.6–10.7	< 0.001	5.9	3.4–10.3	< 0.001
Hypouricemia (pooled)	5.1	3.6–7.4	< 0.001	6.0	4.1–8.8	< 0.001	5.6	3.8–8.3	< 0.001

	Univariate			Multivariate—model 1			Multivariate—model 2		
	sHR	95% CI	*P*	Adj. sHR	95% CI	*P*	Adj. sHR	95% CI	*P*
*Competing risks (mechanical ventilation)*									
No hypouricemia	1.0 (ref.)	–	–	1.0 (ref.)	–	–	1.0 (ref.)	–	–
Hypouricemia (discovery)	11.1	5.2–23.7	< 0.001	17.9	7.3–43.7	< 0.001	7.8	3.1–20.0	< 0.001
Hypouricemia (validation)	41.6	12.6–137.5	< 0.001	40.2	12.0–134.9	< 0.001	36.4	10.6–124.9	< 0.001
Hypouricemia (pooled)	18.1	9.7–33.8	< 0.001	20.8	10.4–41.4	< 0.001	18.8	9.3–38.1	< 0.001

Univariate and multivariate Cox regression analyses predicting mechanical ventilation or death and competing risk regression analyses predicting need for mechanical ventilation taking into account the competing risks of death or discharge. Model 1 is adjusted for age, gender and baseline biological parameters (CRP, LDH, lymphocytes). Model 2 is adjusted for the same parameters and severity biological parameters (higher CRP and LDH levels) during hospitalization

HR, hazard ratio; sHR, subdistribution HR; adj., adjusted; 95% CI, 95% confidence interval

The 59 (11%) patients who died without mechanical ventilation were elderly individuals (median age [IQR]: 83 years [75–89]) with severe comorbidities and/or disabilities (cardiovascular disease, 61%; chronic kidney disease, 32%; nursing home residents, 42%), for whom major therapeutic restrictions had been set.

### Expression of urate transporter URAT1 in the kidney proximal tubules

Post-mortem examination of kidneys from patients with COVID-19 showed structural alterations in the proximal tubule and decreased expression of the multi-ligand receptor megalin, which mediates the reabsorption of low molecular weight proteins [8]. Based on these observations and on the defective tubular handling of uric acid, we investigated the expression of urate transporter URAT1 (SLC22A12), which mediates the reabsorption of uric acid at the apical membrane of proximal tubule cells in kidneys from COVID-19 patients (Additional file 1: Table S3).

In a small series of 5 kidney autopsy samples from patients who died of COVID-19, confocal microscopy suggested a reduction in the expression of URAT1 as compared to control kidneys (mean relative maximal intensity 0.21 ± 0.12 vs. 1.00 ± 0.27, *P* = 0.001) (Additional file 1: Fig. S3). In contrast, the expression of aquaporin-1, used as an internal control for the proximal tubule brush border, was not different between cases and controls (0.75 ± 0.47 vs. 1.00 ± 0.47, *P* = 0.5). The median [IQR] lowest level serum uric acid in these patients was 2.3 mg/dl [1.5–3.8].

In line with previous evidence showing defective tubular handling of uric acid, these observations suggest that altered expression of urate transporters in the kidney proximal tubule contributes to the development of hypouricemia in patients with COVID-19.

## Discussion

Using two independent cohorts, we showed that low serum levels of uric acid are common among patients hospitalized for SARS-CoV-2 infection and closely associate with disease severity and with the need of mechanical ventilation. Thorough assessment of the kinetics of serum uric acid levels showed an acute and reversible decrease that parallels the development of respiratory failure. The occurrence of hypouricemia was independent from drugs interfering with uric acid production, nephrotoxic medications, treatment received for COVID-19 or viral load, but was associated with defective tubular handling of uric acid and pathological features suggestive of an altered expression of urate transporter in the kidney proximal tubule.

The prevalence of hypouricemia is approximately 0.3% in the general ambulatory population and ranges between 1.2% and 2.5% among hospitalized patients [15, 16]. In our study, 20% of the patients hospitalized for SARS-CoV-2 infection developed hypouricemia, a proportion that increased to 77% among patients requiring mechanical ventilation. The association between hypouricemia and progression to respiratory failure requiring mechanical ventilation was independent from age, gender, comorbidities and biological parameters of disease severity, suggesting its potential use as a biomarker to identify patients at risk of more severe COVID-19. The high prevalence of hypouricemia in COVID-19 patients is reminiscent of that observed during the 2003 outbreak of SARS, where ~ 25% of patients with SARS-CoV infection developed hypouricemia, with higher rates in patients requiring mechanical ventilation [10]. These findings are also in line with recent data from retrospective cohorts of Chinese patients with COVID-19, in whom low serum levels of uric acid were associated with more severe symptoms [17–19] and with an increased risk of death [19].

Uric acid is the end-product of purine metabolism in humans, who lack uricase, and is generated in the liver, a process catalyzed by xanthine oxidase. The kidney is an important regulator of circulating uric acid levels as it excretes most of total body uric acid [20]. Serum urate is freely filtered by the glomeruli followed by a complex balance of reabsorption and secretion in the kidney proximal tubule. Although the molecular mechanisms of urate transport in the proximal tubule are still incompletely understood, URAT1 (SLC22A12) is the main apical transporter mediating urate reabsorption in the brush border of the proximal tubule [21]. Its major role was further supported by the strong association between *SLC22A12* gene variants and serum uric acid levels in the general population [22]. Defective tubular handling of urate contributes to the development of hypouricemia in patients with life-threatening COVID-19, a mechanism that is supported by the presence of inappropriate uricosuria and by the association with other features of proximal tubule dysfunction [8]. In a small subset of kidney samples from patients who died from COVID-19, we showed that life-threatening SARS-CoV-2 infection is associated with a significant (~ 70%) decrease in the expression of the apical urate transporter URAT1 in the kidney proximal tubule, contributing to the impaired tubular absorption of urate. Of note, experimental data showed that acute inflammation elicited by viral mimetics in rats downregulates the expression of kidney tubule transporters, including URAT1 [23]. Upstream mechanisms linking viral infection and downregulation of proximal tubule transporters remain to be elucidated, but may involve either direct viral cytotoxic effects on the proximal tubule and/or indirect effects resulting from pro-inflammatory cytokines [24]. Although we found no association with the use of urate lowering drugs or severe liver disease, we cannot formally rule out that impaired uric acid generation, i.e., through reduced dietary intake, may have contributed to aggravate hypouricemia among the sickest patients. We also found no evidence for the presence of the syndrome of inappropriate anti-diuretic hormone secretion in patients with life-threatening COVID-19 and hypouricemia, nor for any massive fluid infusion potentially causing fluid overload and hemodilution. None of the patients included in our study received parenteral nutrition.

The mechanisms linking hypouricemia and progression to severe disease requiring mechanical ventilation in patients with COVID-19 remains speculative and may be diverse. First, studies have shown uric acid to be an important antioxidant [25] and poor outcomes in patients with hypouricemia might be related to reduced defense against oxidative stress. Second, uric acid was found to act as an endogenous modulator of innate immunity, and it is tempting to speculate that low levels of serum uric acid may therefore exacerbate the cytokine storm observed during COVID-19 [26, 27]. Third, recent evidence demonstrated that acute and severe hypouricemia induced in healthy individuals causes endothelial dysfunction, reduces blood pressure, decreases myeloperoxidase activity and increases lipid peroxidation [28]. Lastly, as proximal tubule cells and pneumocytes both express angiotensin-converting enzyme 2 (ACE2), the cellular receptor mediating viral entry into host cells, we cannot rule out a parallel evolution at the tubular and lung level, resulting from putative direct cellular infection by SARS-CoV-2 in both organs [8]. The potential contribution of each of these factors to the relationship between low levels of serum uric acid and poor outcomes in patients with COVID-19 will require further investigation.

The strengths of this study include the availability of large and well-characterized discovery and validation cohorts; detailed kinetics of serum uric acid; robust outcome analysis; and availability of post-mortem kidney samples for expression studies. We also acknowledge limitations, including the single center design of the study; the lack of mechanistic insights into the pathophysiology linking hypouricemia and respiratory failure; and absence of systematic longitudinal follow-up for all biological parameters.

## Conclusions

In summary, acute and severe hypouricemia is highly prevalent among patients with COVID-19 requiring hospitalization and is independently associated with disease severity and with progression toward respiratory failure requiring mechanical ventilation. These data suggest that serum uric acid could be used as a reliable biomarker to identify patients at risk of life-threatening COVID-19.

## Supplementary Information

**Additional file 1: Figure S1:** Progression of severity during the course of the disease in the discovery cohort, according to the absence or presence of hypouricemia. **Figure S2:** Kinetics of serum uric acid levels in patients requiring mechanical ventilation in the discovery cohort. **Figure S3:** Expression of urate transporter URAT1 in the kidney proximal tubules. **Table S1:** Characteristics of patients with vs. without available serum levels of uric acid in the discovery cohort. **Table S2:** Baseline characteristics of patients from the discovery cohort, stratified for the absence or presence of hypouricemia. **Table S3:** Characteristics of COVID-19 and control patients with kidney samples used for expression studies.

## Data Availability

The datasets used and analyzed during the current study are available from the corresponding author on reasonable request.

## Abbreviations

ACE: Angiotensin-converting enzyme
CI: Confidence interval
COVID-19: Coronavirus disease 2019
HR: Hazard ratio
hsCRP: Highly sensitive C-reactive protein
IQR: Interquartile range
KDIGO: Kidney Disease Improving Global Outcomes
LDH: Lactate dehydrogenase
RT-PCR: Reverse transcription polymerase chain reaction
sHR: Subdistribution hazard ratio
