# Supplementary material for: Serum uric acid, disease severity and outcomes in COVID-19
Source: Crit Care. 2021 Jun 14;25:212. doi: 10.1186/s13054-021-03616-3 (PMC8201458; doi:10.1186/s13054-021-03616-3)
Supplement: Supplementary file 1 — Additional file 1: Figure S1: Progression of severity during the course of the disease in the discovery cohort, according to the absence or presence of hypouricemia. Figure S2: Kinetics of serum uric acid levels in patients requiring mechanical ventilation in the discovery cohort. Figure S3: Expression of urate transporter URAT1 in the kidney proximal tubules. Table S1: Characteristics of patients with vs. without available serum levels of uric acid in the discovery cohort. Table S2: Baseline characteristics of patients from the discovery cohort, stratified for the absence or presence of hypouricemia. Table S3: Characteristics of COVID-19 and control patients with kidney samples used for expression studies. [file 13054_2021_3616_MOESM1_ESM.docx]

***Supplementary Information***

**Serum Uric Acid, Disease Severity and Outcomes in COVID-19**

*Inès Dufour, Alexis Werion et al.*

**Table of content**

**Figure S1:** Progression of severity during the course of the disease in the discovery cohort, according to the absence or presence of hypouricemia.

**Figure S2:** Kinetics of serum uric acid levels in patients requiring mechanical ventilation in the discovery cohort.

**Figure S3:** Expression of urate transporter URAT1 in the kidney proximal tubules.

**Table S1:** Characteristics of patients with vs. without available serum levels of uric acid in the discovery cohort.

**Table S2:** Baseline characteristics of patients from the discovery cohort, stratified for the absence or presence of hypouricemia.

**Table S3:** Characteristics of COVID-19 and control patients with kidney samples used for expression studies.

**Figure S1. Progression of severity during the course of the disease in the discovery cohort, according to the absence or presence of hypouricemia.**

**
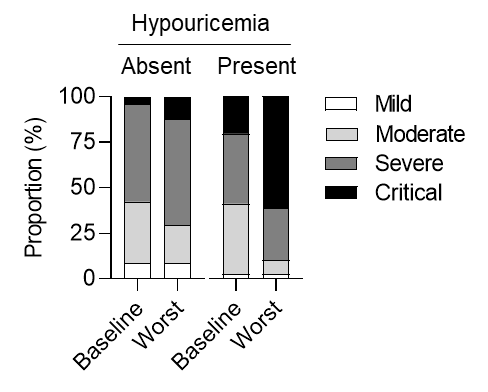
**

**Figure S2. Kinetics of serum uric acid levels in patients requiring mechanical ventilation in the discovery cohort.**

**
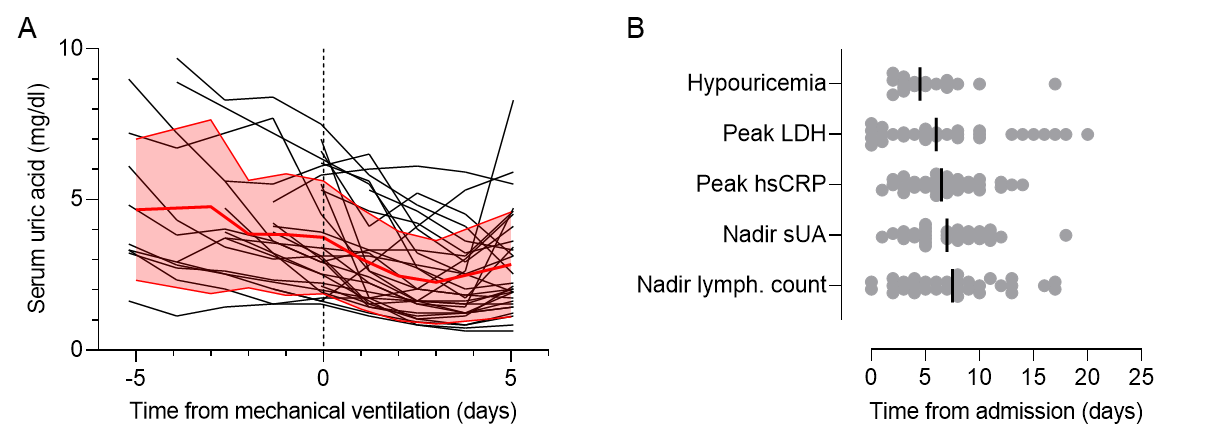
**

(**A)** Kinetics of serum acid in relation to the clinical course of respiratory failure and mechanical ventilation initiation. Black lines represent individual patients, and red lines are the mean and standard deviation. (**B**) Time from admission to the peak of lactate dehydrogenase (LDH), onset of hypouricemia, nadir lymphocytes count and peak of highly sensitive C-reactive protein (hsCRP) in the subset of patients requiring mechanical ventilation (n=32).

**Figure S3. Expression of urate transporter URAT1 in the kidney proximal tubules.**

**
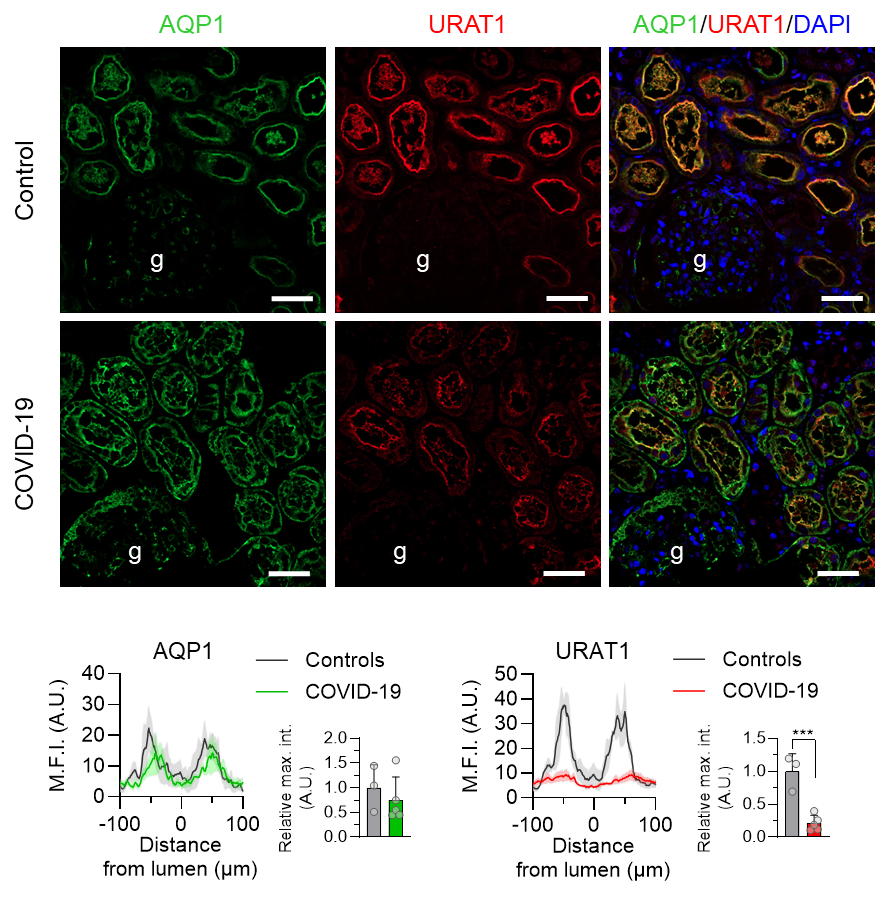
**

Representative pictures of double immunostaining with anti-AQP1 (green channel) and anti-URAT1 (red channel) antibodies viewed under confocal fluorescence microscopy in kidneys sections from a control and a patient with COVID-19. Nuclei are stained with DAPI (blue channel). Original magnification, 20x. Bars, 50 μm. Mean fluorescence intensity (M.F.I.) profiles and relative maximal intensity (rel. max. int.) for AQP1 and URAT1 were quantified on cross-sectional sections of proximal tubules from 3 controls (grey) and 5 patients who died of COVID-19 (green for AQP1 and red for URAT1) using ZEN. Data are mean values and s.e.m. ***P=0.001, unpaired t-test.

**Table S1. Characteristics of patients with vs. without available serum levels of uric acid.**

| **Demographics and comorbidities** | **Whole cohort n=215** | **Uric acid available n=192** | **Uric acid unavailable n=23** |
| --- | --- | --- | --- |
| Age, median (IQR), years | 67 (56-81) | 65 (55-79) | 80 (64-91) |
| Male gender – no. (%) | 117 (54) | 106 (55) | 11 (48) |
| Ethnicity – no. (%) |  |  |  |
| Caucasian | 180 (84) | 162 (84) | 18 (78) |
| Sub-Saharan African | 31 (14) | 27 (14) | 4 (17) |
| Other | 4 (2) | 3 (2) | 1 (4) |
| Cardiovascular disease – no. (%) | 49 (23) | 44 (23) | 5 (22) |
| Chronic kidney disease – no. (%) | 43 (20) | 39 (20) | 5 (22) |
| Hypertension – no. (%) | 105 (49) | 97 (51) | 8 (35) |
| Diabetes – no. (%) | 51 (24) | 46 (24) | 5 (22) |
| Human immunodeficiency virus infection – no. (%) | 3 (1) | 3 (2) | 0 (0) |
| Chronic liver disease – no. (%) | 5 (2) | 5 (3) | 0 (0) |
| Chronic pulmonary disease – no. (%) | 21 (10) | 19 (10) | 2 (9) |
| Medications – no. (%) |  |  |  |
| Allopurinol or febuxostat | 12 (6) | 11 (6) | 1 (4) |
| Angiotensin receptor blocker | 31 (14) | 30 (16) | 1 (4) |
| ACE inhibitor | 43 (20) | 39 (20) | 4 (17) |
| Chronic immunosuppressive treatment^a^ | 16 (7) | 16 (8) | 0 (0) |
| Anti-cancer drugs^b^ | 9 (4) | 9 (5) | 0 (0) |
| **Symptoms and vitals at admission** |  |  |  |
| Duration of symptoms, median (IQR), days | 6 (2-8) | 6 (2-8) | 5 (1-10) |
| Symptoms at admission – no. (%) |  |  |  |
| Fever | 165/214 (77) | 150/191 (79) | 15 (65) |
| Cough | 140/213 (66) | 127/190 (67) | 13 (57) |
| Dyspnea | 148/213 (69) | 133/190 (70) | 15 (65) |
| Sore throat | 24/213 (11) | 20/190 (11) | 4 (17) |
| Confusion | 27/213 (13) | 21/190 (11) | 6 (26) |
| Anosmia/agueusia | 19/213 (9) | 18/190 (9) | 1 (4) |
| Rhinitis | 38/213 (18) | 34/190 (18) | 4 (17) |
| Diarrhea | 50/213 (23) | 42/190 (22) | 8 (35) |
| Chest pain | 14/213 (7) | 13/190 (7) | 1 (4) |
| Sa02, median (IQR), % | 91 (88-95) | 91 (88-95) | 90 (86-94) |
| Systolic BP, median (IQR), mmHg | 134 (121-149) | 134 (123-150) | 130 (113-146) |
| Diastolic BP, median (IQR), mmHg | 77 (68-84) | 77 (69-84) | 73 (63-92) |
| Heart rate, median (IQR), bpm | 90 (82-102) | 91 (82-103) | 85 (72-96) |
| **Lab tests, dipstick and chest CT** |  |  |  |
| hsCRP, median (IQR), mg/l | 77 (39-130) | 74 (38-130) | 87 (53-136) |
| Glycemia, median (IQR), mg/dl | 119 (105-139) | 120 (106-144) | 111 (101-124) |
| Serum creatinine, median (IQR), mg/dl | 1.0 (0.8-1.2) | 1.0 (0.8-1.2) | 1.1 (0.8-1.3) |
| eGFR, median (IQR), ml/min/1.73 m2 | 71 (49-84) | 72 (50-85) | 57 (38-78) |
| Serum uric acid, median (IQR), mg/dl | 4.8 (3.7-6.2) | 4.8 (3.7-6.2) | - |
| Sodium, median (IQR), mmol/l | 137 (134-140) | 137 (134-140) | 137 (136-141) |
| Bicarbonate, median (IQR), mmol/l | 24 (22-26) | 24 (22-26) | 26 (22-28) |
| AST, median (IQR), IU/l | 36 (27-59) | 36 (26-54) | 40 (30-84) |
| ALT, median (IQR), IU/l | 27 (17-44) | 27 (17-43) | 23 (17-44) |
| Total bilirubin, median (IQR), mg/dl | 0.5 (0.4-0.6) | 0.5 (0.4-0.7) | 0.5 (0.4-0.6) |
| CK, median (IQR), IU/l | 116 (65-293) | 111 (62-241) | 204 (86-427) |
| LDH, median (IQR), IU/l | 353 (272-452) | 349 (270-449) | 389 (306-479) |
| Lymphocytes, median (IQR), n/µl | 840 (600-1160) | 830 (590-1150) | 860 (620-1270) |
| Platelets, median (IQR), 10^3^/µl | 204 (139-254) | 202 (136-258) | 225 (172-249) |
| Dipstick proteinuria – no. (%) |  |  |  |
| 0 | 36/169 (21) | 33/151 (22) | 3/18 (17) |
| 1+ | 55/169 (33) | 49/151 (32) | 6/18 (33) |
| 2+ | 68/169 (40) | 59/151 (39) | 9/18 (50) |
| 3+ | 10/169 (6) | 10/151 (7) | 0/18 (0) |
| **Computed tomography scan of the chest upon admission** | | | |
| Extent of lesions on chest CT scan – no. (%) |  |  |  |
| <10% | 26/173 (15) | 24/156 (15) | 2 (12) |
| 10-25% | 65/173 (38) | 59/156 (38) | 6 (35) |
| 25-50% | 54/173 (31) | 48/156 (31) | 6 (35) |
| >50% | 28/173 (16) | 25/156 (16) | 3 (18) |
| **Drugs received for COVID-19** |  |  |  |
| Hydroxychloroquine – no. (%) | 177 (82) | 164 (85) | 13 (57) |
| Azithromycin – no. (%) | 24 (11) | 21 (11) | 3 (13) |
| Anti-viral drugs^c^ – no. (%) | 2 (1) | 2 (1) | 0 (0) |
| Immunomodulatory drugs^d^ - no. (%) | 30 (14) | 28 (15) | 2 (9) |
| **Outcomes** |  |  |  |
| Follow-up, median (IQR), days | 123 (49-130) | 123 (107-129) | 116 (6-131) |
| Death – no. (%) | 54 (25) | 43 (22) | 11 (48) |
| Mechanical ventilation – no. (%) | 32 (15) | 32 (17) | 0 |
| Acute kidney injury – no. (%) | 23 (11) | 22 (11) | 1 (4) |
| Kidney replacement therapy – no. (%) | 6 (3) | 6 (3) | 0 |
| Hospital length of stay, median (IQR), days | 11 (6-17) | 12 (7-18) | 6 (4-11) |

^a^Chronic immunosuppressive treatment included (one or more per patient) ciclosporin A (1), corticosteroids (10), methotrexate (2), rituximab (1), etanercept (1), mycophenolate mofetil (1), ocrelizumab (1), tocilizumab (2). ^b^Anti-cancer drugs included cyclophosphamide (1), doxorubicine (1), vincristine (1), venetoclax (1), cisplatin (2), cytarabine (1), axitinib (1), bortezomib (2), thalidomide/pomalidomide (2), paclitaxel (1). ^c^Antiviral drugs included favipiravir (1) and lopinavir (1). ^d^Immunomodulatory drugs for COVID-19 (one or more per patient) included corticosteroids (18), interleukin-7 (11), tocilizumab (1). Continuous variables are expressed as median and interquartile range (IQR), and categorical variables as numbers (no.) and percentages (%). Sa02, oxygen saturation while breathing ambient air; BP, blood pressure; eGFR, CKD-EPI estimated glomerular filtration rate; hsCRP, highly-sensitive C-reactive protein; AST, aspartate aminotransferase; ALT, alanine aminotransferase; CK, creatine kinase; LDH, lactate dehydrogenase; CT, computed tomography.

**Table S2. Baseline characteristics of patients, stratified for the absence or presence of hypouricemia, in the discovery cohort.**

| **Demographics and comorbidities** | **Overall**  **n=192** | **No hypouricemia n=153** | **Hypouricemia n=39** | **P** |
| --- | --- | --- | --- | --- |
| Age, median (IQR), years | 65 (55-79) | 66 (56-82) | 63 (53-72) | 0.1 |
| Male gender – no. (%) | 106 (55) | 87 (57) | 19 (49) | 0.4 |
| Ethnicity – no. (%) |  |  |  | 0.2 |
| Caucasian | 162 (84) | 131 (86) | 31 (79) |  |
| Sub-Saharan African | 27 (14) | 21 (14) | 6 (15) |  |
| Other | 3 (2) | 1 (1) | 2 (5) |  |
| Cardiovascular disease – no. (%) | 44 (23) | 40 (26) | 4 (10) | 0.04 |
| Chronic kidney disease – no. (%) | 39 (20) | 36 (24) | 3 (8) | 0.03 |
| Hypertension – no. (%) | 97 (51) | 78 (51) | 19 (49) | 0.8 |
| Diabetes – no. (%) | 46 (24) | 39 (25) | 7 (18) | 0.3 |
| Medications – no. (%) |  |  |  |  |
| Allopurinol or febuxostat | 11 (6) | 8 (5) | 3 (8) | 0.6 |
| Fenofibrate | 3 (2) | 2 (1) | 1 (3) | 0.6 |
| Angiotensin receptor blocker | 30 (16) | 24 (16) | 6 (15) | 0.9 |
| ACE inhibitor | 39 (20) | 29 (19) | 10 (26) | 0.4 |
| Chronic immunosuppressive treatment^a^ | 16 (8) | 11 (7) | 5 (13) | 0.3 |
| Anti-cancer drugs^b^ | 9 (5) | 5 (3) | 4 (10) | 0.07 |
| **Symptoms and vitals at admission** |  |  |  |  |
| Duration of symptoms, median (IQR), days | 6 (2-8) | 6 (2-8) | 7 (3-8) | 0.9 |
| Symptoms at admission – no. (%) |  |  |  |  |
| Fever | 150/191 (79) | 118/150 (78) | 32/39 (82) | 0.6 |
| Cough | 127/190 (67) | 103/151 (58) | 24/39 (62) | 0.4 |
| Dyspnea | 133/190 (70) | 107/151 (71) | 26/39 (67) | 0.6 |
| Diarrhea | 42/190 (22) | 30/151 (20) | 12/39 (31) | 0.1 |
| Chest pain | 13/190 (7) | 9/151 (6) | 4/39 (10) | 0.3 |
| Admission via emergency department – no. (%) | 186 (97) | 149 (97) | 37 (95) | 0.4 |
| Sa02, median (IQR), % | 91 (88-95) | 91 (88-95) | 92 (83-95) | 0.4 |
| **Lab tests at admission** |  |  |  |  |
| hsCRP, median (IQR), mg/l | 74 (38-130) | 70 (34-126) | 105 (56-170) | 0.05 |
| Creatinine, median (IQR), mg/dl | 1.0 (0.8-1.2) | 1.0 (0.9-1.3) | 0.9 (0.7-1.1) | 0.005 |
| eGFR, median (IQR), ml/min/1.73 m2 | 72 (50-85) | 70 (47-82) | 75 (67-94) | 0.004 |
| Serum uric acid, median (IQR), mg/dl | 4.8 (3.7-6.2) | 5.2 (4.0-6.5) | 3.3 (2.2-4.2) | <0.001 |
| Sodium, median (IQR), mmol/l | 137 (134-140) | 137 (134-140) | 136 (133-138) | 0.3 |
| Bicarbonates, median (IQR), mmol/l | 24 (22-26) | 24 (23-26) | 24 (22-26) | 0.7 |
| LDH, median (IQR), IU/l | 349 (270-449) | 341 (268-440) | 372 (272-542) | 0.2 |
| Lymphocytes, median (IQR), µl^-1^ | 830 (590-1150) | 860 (620-1150) | 700 (500-1130) | 0.08 |
| Platelets, median (IQR), 10^3^/µl | 202 (136-258) | 197 (137-251) | 205 (130-292) | 0.5 |
| Dipstick proteinuria – no. (%) | 118 (61) | 96/121 (79) | 22/30 (73) | 0.7 |
| Viral load on nasopharyngeal swab, median (IQR), Ct | 31 (26-35) | 31 (26-35) | 29 (26-35) | 0.4 |
| **Chest CT scan at admission** |  |  |  |  |
| Extent of lesions– no. (%) |  |  |  |  |
| <10% | 24/156 (15) | 19/125 (15) | 5/31 (16) |  |
| 10-25% | 59/156 (38) | 50/125 (40) | 9/31 (29) |  |
| 25-50% | 48/156 (31) | 39/125 (31) | 9/31 (29) |  |
| >50% | 25/156 (16) | 17/125 (14) | 8/31 (26) |  |
| **Drugs received for COVID-19** |  |  |  |  |
| Hydroxychloroquine – no. (%) | 164 (85) | 127 (83) | 37 (95) | 0.06 |
| Azithromycin – no. (%) | 21 (11) | 17 (11) | 4 (10) | 0.9 |
| Anti-viral drugs^c^ – no. (%) | 2 (1) | 2 (1) | 0 (0) | 0.8 |
| Immunomodulatory drugs^d^ - no. (%) | 28 (15) | 18 (12) | 10 (26) | 0.03 |

^a^Chronic immunosuppressive treatment included (one or more per patient) ciclosporin A (1), corticosteroids (10), methotrexate (2), rituximab (1), etanercept (1), mycophenolate mofetil (1), ocrelizumab (1), tocilizumab (2). ^b^Anti-cancer drugs included (one or more per patient) cyclophosphamide (1), doxorubicine (1), vincristine (1), venetoclax (1), cisplatin (2), cytarabine (1), axitinib (1), bortezomib (2), thalidomide/pomalidomide (2), paclitaxel (1). ^c^Antiviral drugs included favipiravir (1) and lopinavir (1). ^d^Immunomodulatory drugs for COVID-19 (one or more per patient) included corticosteroids (18), interleukin-7 (11), tocilizumab (1). Continuous variables are expressed as median and interquartile range (IQR), and categorical variables as numbers (no.) and percentages (%). Sa02, oxygen saturation while breathing ambient air; BP, blood pressure; eGFR, CKD-EPI estimated glomerular filtration rate; hsCRP, highly-sensitive C-reactive protein; LDH, lactate dehydrogenase; CT, computed tomography.

**Table S3. Characteristics of COVID-19 and control patients with kidney samples used for expression studies.**

| **Condition** | **Age  (yrs)** | **Gender** | **Lowest value of sUA (mg/dL)** | **Need of vaso-**  **pressors** | **UPCR (g/g)** | **AKI/KRT** | **MV** | **LOS before death (days)** | **IFTA (%)** | **ATN** | **Loss of brush border** |
| --- | --- | --- | --- | --- | --- | --- | --- | --- | --- | --- | --- |
| COVID-19 | 74 | M | 1.4 | + | 0.7 | +/- | + | 11 | 1-25 | + | + |
| COVID-19 | 71 | F | 1.1 | + | 1.5 | -/- | + | 20 | 1-25 | + | - |
| COVID-19 | 64 | M | 1.7 | - | 1.5 | -/- | + | 12 | 1-25 | + | + |
| COVID-19 | 57 | M | 2.9 | + | NA | +/+ | + | 21 | 1-25 | + | + |
| COVID-19 | 60 | M | 4.1 | + | 3.3 | +/+ | + | 37 | 1-25 | + | + |
| Membranous nephropathy | 59 | M | NA | - | 8.0 | -/- | - | - | 1-25 | + | + |
| Implantation kidney biopsy | 51 | M | NA | NA | NA | -/- | + | NA | 1-25 | + | - |
| Cardiogenic shock | 46 | M | 5.2 | + | 0.4 | +/+ | + | 6 | NA | + | + |

AKI, acute kidney injury; ATN, acute tubular necrosis; F, female; HTN, hypertension; IFTA, interstitial fibrosis and tubular atrophy; KRT, kidney replacement therapy; M, male; MV, mechanical ventilation; LOS, length of stay; Scler., sclerotic (globally); sUA, serum uric acid; UPCR, urinary protein to creatinine ratio; NA, not available.
